# Switchable Adhesion Surfaces with Enhanced Performance Against Rough Counterfaces

**DOI:** 10.3390/biomimetics1010002

**Published:** 2016-02-25

**Authors:** Lizbeth O. Prieto-López, John A. Williams

**Affiliations:** 1 Department of Engineering, University of Cambridge, Trumpington Street, Cambrige CB2 1PZ, UK; Lizbeth.Prieto@leibniz-inm.de; 2 Robinson College, University of Cambridge, Cambridge CB3 9AN, UK

**Keywords:** switchable adhesion, soft adhesion, microfluidics, roughness

## Abstract

In a recent study, we demonstrated that the pressurization of micro-fluidic features introduced in the subsurface of a soft polymer can be used to actively modify the magnitude of the adhesion to a harder counterface by changing its waviness or long wavelength undulations. In that case, both contacting surfaces had very smooth finishes with root-mean-square roughnesses of less than 20 nm. These values are far from those of many engineering surfaces, which usually have a naturally occurring roughness of between ten and a hundred times this value. In this work, we demonstrate that appropriate surface features, specifically relatively slender “fibrils”, can enhance the ability of a such a soft surface to adhere to a hard, but macroscopically rough, counterface, while still maintaining the possibility of switching the adhesion force from one level to another. Conversely, stiffer more conical surface features can suppress adhesion even against a smooth counterface. Examples of each form of topography can be found in the natural world.

## 1. Introduction

In a previous work, the development of soft surfaces with controllable adhesion was presented [1]; this was brought about by the pressurization of subsurface chambers, which allowed a switch in the magnitude of the adhesion force against a harder counterface from one level to another by modifying the soft surface’s waviness or long wavelength undulations. In that study, both the surfaces participating in the contact were nominally smooth with root-mean-square or roughnesses of less than 20 nm. These are very much lower roughness values than those of conventionally engineered metallic surfaces, which will typically have roughnesses of between ten and hundred times this figure.

The extent of adhesion between a hard and a smooth soft material is greatly reduced by any roughness on the harder surface. This is because of the very short-range nature (only nanometres) of the van der Waal forces on which adhesion depends. To be effective there must be intimate contact between the two surfaces. When the hard surface is insufficiently smooth, the soft material has to deform elastically to conform to the detailed topography of the counterface and this stores additional elastic energy within the soft material. When the external load is reduced or removed, this stored energy becomes available to peel apart any adhered junctions that have been formed and thus destroy the observation of macroscopic adhesion. It is primarily for this reason that adhesion between macroscopic objects is not encountered in everyday life. Paradoxically, in order to maintain adhesion with a rough hard surface it is necessary to introduce some texture onto the softer surface so the local accommodation to the counterface topography becomes possible with a reduced elastic energy penalty and, thus, improved maintenance of macroscopic adhesion.

Nature has mastered the task of providing “good” adhesion between naturally rough surfaces by developing features with distributed structural stiffness. Hierarchical fibrillar pads represent a frequently found natural solution for situations in which a strong adhesive force is required which must be highly dynamic and effective over a wide range of surface roughnesses: examples are the feet of geckos [2] and of insects, such as spiders and beetles [3]. The physical principle behind the versatility of these fibrillar pads lies in the hierarchical compliance in the outermost regions of their structure. This allows the deformation of their surface to conform to the counter-surface topography with the minimum storage of elastic energy. This ensures a large area in intimate contact that can collectively provide a greater energy of adhesion than the elastic energy stored due to the deformation of the surface [4,5]. Taking inspiration from these natural structures for enhanced adhesion against rough counterfaces, we have introduced a “brush-like” texture on the surfaces of the switchable adhesion devices. Once again, increasing the long wavelength waviness in the contact, through a network of pressurized buried chambers, will peel the surfaces apart.

## 2. Materials and Methods

### 2.1. Specimen Fabrication

**Figure 1:**
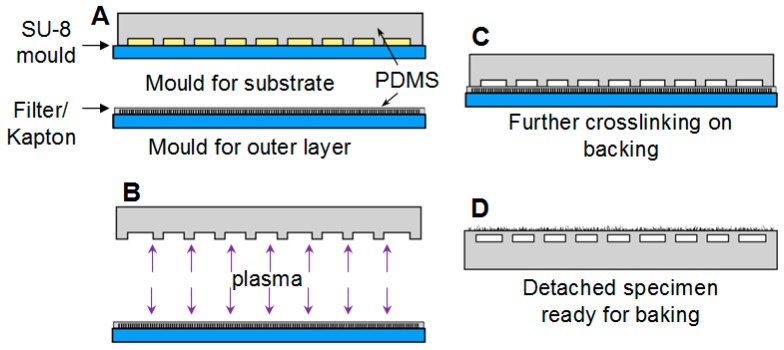
Fabrication process for switchable adhesion devices with textured surfaces. (**A**) Moulding substrate and outer layers; (**B**) plasma activation of surfaces prior to bonding; (**C**) bonding basic structure of PDMS to the skin layer still supported on the glass backing; (**D**) specimen detached from the glass substrate, ready for baking.

**Figure 2:**
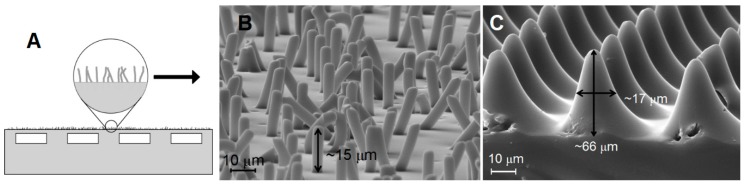
Examples of textured outer surfaces generated by casting PDMS. (**A**) Diagrammatic side view of a specimen with textured surface; (**B**) Surface casted against Millipore Track etched Isopore polycarbonate membrane filter with 5 µm pore size, surface labelled as “fibrillar”; and (**C**) laser ablated Kapton film labelled as “conical pillars”.

The fabrication of the devices followed a similar protocol to that described in Reference [1], which is based on standard micro-fluidics protocols. The textured finish was introduced on the surface of the specimens by an additional step consisting of casting the outer layer against a suitable mould. The fabrication process is illustrated in Figure 1. Two different surface features were obtained from different types of mould. An array of randomly oriented slender pillar-like features was fabricated using Millipore track etched Isopore polycarbonate membrane filter of 5-µm pore size while a much more regular pattern of almost conical pillars was obtained from a laser ablated Kapton film (we are grateful to Professor R. S. Fearing at UC Berkeley for providing the Kapton mould for this sample). SEM images of both surfaces are shown in Figure 2. Sylgard 184 in a base-to-catalyst ratio 10:1 by weight was used as the constituting material of these devices.

### 2.2. Adhesion Measurements

**Figure 3:**
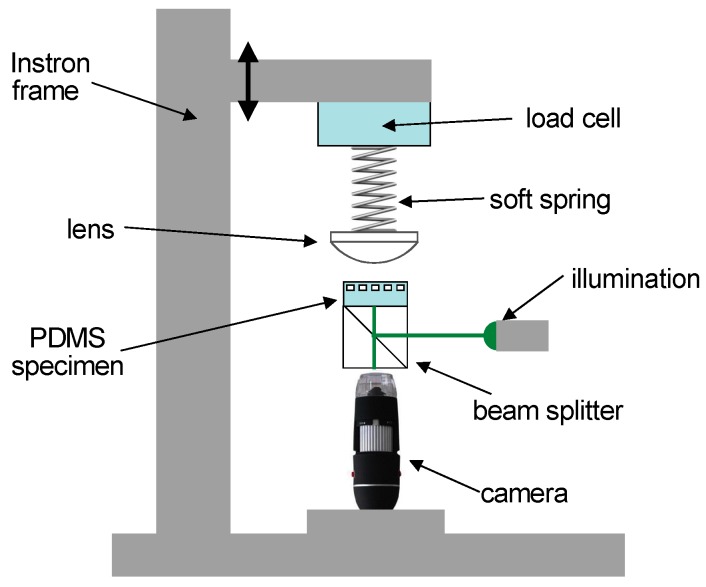
Experimental set-up for adhesion measurements when using the soft spring.

The adhesion properties of the switchable adhesion devices with textured surface for enhanced performance against rough counterfaces were evaluated by measuring the pull-off force, *P*_po_, generated by contact with the surface of a spherical glass lens with a radius of curvature of *R* = 17.97 mm. Two spherical lenses with similar radius of curvature but different surface roughness were used in these measurements: one nominally smooth with a root mean square roughness *R_q_* of *ca.* 19 nm and another roughened by sand blasting to an *R_q_* value of *ca.* 3.7 µm, both measured by a suitable stylus instrument (Form Talysurf i120, Taylor Hobson, Leicester, UK). The tests were carried out in a single column electromechanical testing machine (5544, Instron, Buckinghamshire, UK) with a compression/indentation configuration. A load cell (Instron 2530-439) of capacity of 5 N with accuracy better that 0.025% was used in these experiments. Load and displacement were recorded at a sampling frequency of 10 Hz. The area of contact was imaged throughout the tests using a home-made imaging rig fixed to the base of the Instron. This consisted of a BK7 glass beam-splitter (1 cm × 1 cm × 1 cm, Comar 10JQ01), a digital microscope (AD4113TL-FVW Dino-Lite Premier) with maximum recording speed of 15 fps, and monochrome green 520 nm lighting source (6V LED Edmunds Optics). The lens could be mounted either on a soft, helically wound, polymer spring with stiffness *k* = 15 N m^−1^ or directly to the base of the load cell (stiffness of about *k* = 100 kN m^−1^). The measuring arrangement is illustrated in Figure 3.

## 3. Results and Discussion

**Figure 4:**
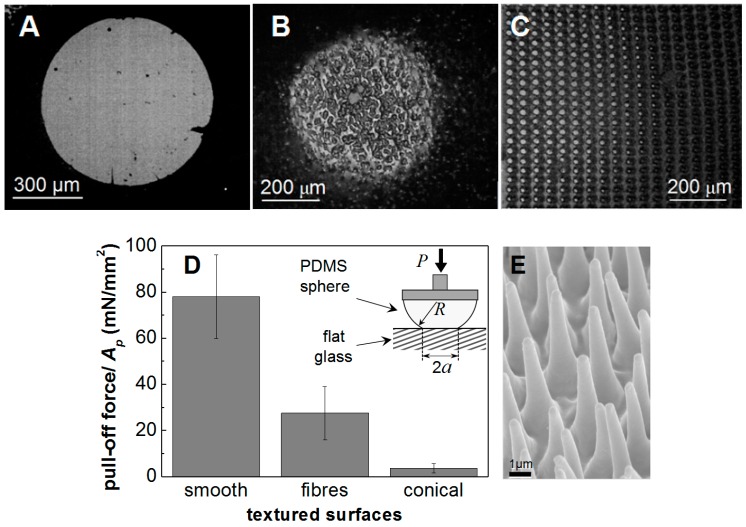
Contact between a smooth and flat glass surface and spherical PDMS lenses with different surface texture under a load of 25 mN. (**A**) Smooth PDMS sphere of *R* = 6.2 mm; (**B**) Fibrillar surface on a PDMS sphere of *R* = 2.1 mm; (**C**) Surface patterned with conical pillars on a PDMS sphere of *R* = 6.2 mm; (**D**) Pull-off force normalized by the Hertzian area of contact; (**E**) A naturally occurring non-adhesive structure—the acanthae of the stick-insect Carausius morosus [7].

A set of preliminary measurements were carried out on the textured surfaces shown in Figure 2. In these, PDMS textured surfaces fixed on spherical PDMS lenses were pressed against the hard flat and smooth (*R_q_* < 10 nm) top surface of the glass beam splitter under a load of 25 mN. The compliant spring was replaced by an effectively rigid link. These experiments allowed a clear visualization of the contact regions formed with each polymeric texture. Parts A, B and C in Figure 4 show images of the contact area against the beam splitter of smooth, fibrillar and conical pillars textures, respectively. In Figure 4D a comparative figure is presented of the pull-off force measured from each surface normalized by the Hertzian contact area, *A_p_*, of a contact with similar load and radius but untextured surfaces. The area is given by *A_p_* = π*a*^2^ where *a* is the radius of contact estimated from the Hertzian expression [6]:

(1) $$a^{3}=RP/K$$

with *R* being the reduced radius of curvature of the conjunction in this case determined by the radius of the PDMS sphere, *P* is the applied load and *K* = 4.6 MPa is the elastic constant of the combined PDMS-glass contact pair. The bars indicate the range of values measured in three experiments.

As anticipated from the significantly larger contact area, the contact with the smooth PDMS surface (Figure 4A) exhibited the highest adhesion force, at 78 mN, among the three types of contact shown in Figure 4. In the case of the textured surfaces, both the reduction in the area available for “true contact” and the energy stored in the elastic hinterland behind the contact spots formed by the higher asperities within the gross contact area, which becomes available to peel apart the adhesive junctions formed by the less prominent asperities, results in the reduction of the adhesion force to values of 23 mN and *ca*. 3 mN, respectively.

The behavior of the fibrillar surface in Figure 2B (contact in Figure 4B) can be contrasted to that of the surface with conical features in Figure 2C (contact in Figure 4C). Although having rather similar tip dimensions, the individual conical pillars will be much more resistant to buckling and so present a much stiffer surface layer than the fibrils of Figure 2B. Consequently they will store more elastic energy in comparison with the similar reduction in surface energy and so would be expected to further reduce the propensity to adhere giving highly reduced values of pull-off force. Experiment shows this to be the case, as shown in Figure 4D. The fibrillar structure retained about 30% of the adhesion of a smooth PDMS surface but the conical pillars required an extremely small pull-off force (<4 mN) compared to the force of *ca.* 80 mN needed to separate a smooth PDMS surface from the same glass counterface.

The requirement for a satisfactory adhesive to exhibit a certain minimum level of compliance to give useful values of “tackiness” has been the subject of various empirical rules in the field of adhesives. The best known being the Dahlquist criterion [8], which states that for success as an adhesive a necessary (though, presumably, not sufficient) condition is that the effective surface modulus *E*_eff_ ≤ 100 kPa. Taking dimensions from the micrographs, Figure 2A,B, and a suitable of value for the modulus of PDMS of about 2.6 MPa, it is possible to estimate the surface compliance of these two morphologies viewed as potential adhesives. Take a mean fibril as having length of 15 µm, diameter 5 µm and inclination to the surface of 10° suggests an individual element compliance of no more than *ca*. 0.55 m N^−1^. Estimating an areal density of fibrils at 2.5 × 10^9^ m^−2^ gives an effective surface modulus of 67 kPa. Analysis, for example, that by Smith [9], indicates that the individual conical peaks of Figure 2C will be much more resistant to buckling than cylindrical pillars of the same tip diameter and material and elastically stiffer by the same factor by which the base diameter exceeds the tip. Taking this ratio as 4 and a slightly lower areal feature density of 1.1 × 10^9^ m^−2^ gives a surface stiffness of these features as 174 kPa. These two values then rather neatly bracket the Dahlquist criterion.

In the same way, there is evidence of natural examples of fibrillar surfaces enhancing adhesion [2,3] so there is for conical protuberances being used to minimize adhesion. Many stick-insects possess tarsal heel pads (euplantulae) covered by arrays of conical, micron sized hairs (acanthae). Figure 4E shows those of the stick-insect Carausius morosus. Acanthae are used mainly under compression and, although are capable of generating high friction, show negligible adhesion. This attribute of their behavior appears to be essential for the pads’ use in locomotion [7].

The data presented thus far were all generated against the very smooth, *R_q_* < 10 nm, hard counterface of the top face of a beam splitter. The situation changes when the hard counterface becomes rougher. Topographies of this sort will exhibit markedly lower adhesion against smooth PDMS due to stored elastic energy within the deformed zone around the contact. This energy balance was explored, inter alia, by Fuller and Tabor [10] who produced a modified adhesion parameter given by:

(2) $$\theta =\frac{E^{*}\sigma^{3/2}R^{1/2}}{Rw}$$

in which σ is the standard deviation of the surface roughness. This non-dimensional group can be interpreted as the ratio of the force needed to elastically indent a block of material with elasticity E* to a depth *R* by a sphere of radius *R* to the potential adhesive force experienced by a sphere of that radius. Significant adhesion requires that θ is small, say θ < 0.1, and adhesion is substantially lost for values of θ > 5. For a glass lens of radius 18 mm, against a block of smooth PDMS, this would correspond to roughness of around 5 µm. This effect was investigated by roughening the surface of a glass lens by sand blasting to a value of 3.7 µm so that its adhesion to both a smooth and a fibrillar surface could be compared to the values obtained from the contact with a nominally smooth glass lens of the similar radius of curvature.

**Figure 5:**
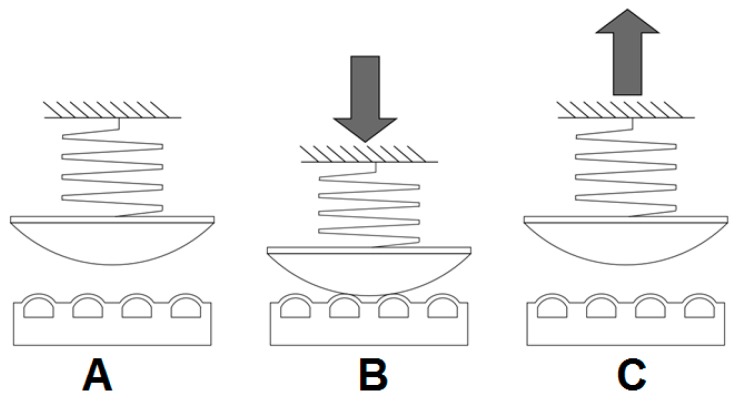
Adhesion experiments. (**A**) Increase pressure at set point before contact; (**B**) contact at 5 mN load for 60 s; (**C**) tensile load until detachment.

This set of adhesion tests followed the sequence illustrated in Figure 5. The PDMS specimen, which could either be planar or initially pressurized to certain level to produce a change in its surface topography, was mounted on the upper surface of the beam splitter, as indicated in Figure 3. The glass lens was then lowered into contact with the specimen at a machine cross-head speed of 5 μm s^−1^, equivalent to a loading rate of 75 μN s^−1^, up to a maximum load of 5 mN. The load was maintained for a dwell time of 60 s after which the lens was withdrawn at a cross-head rate of 5 μm s^−1^ until a critical tensile load *P*_po_ produced detachment. The tensile force required for breaking the contact between the lens and the specimen was again taken as a measure of the adhesive properties of the surface.

**Figure 6:**
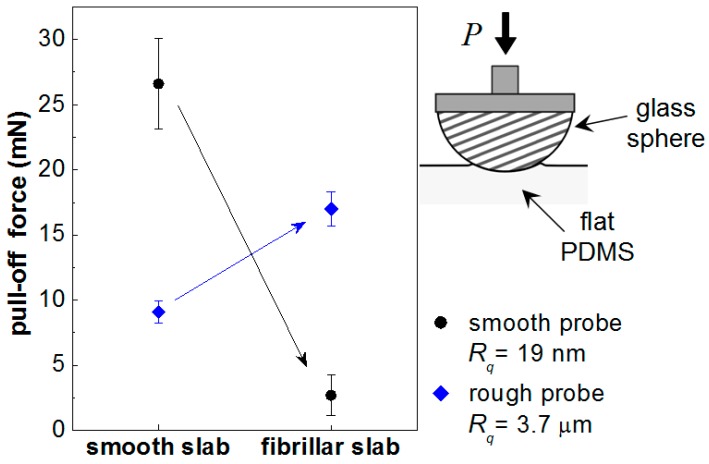
Pull-off force measurements between flat PDMS surfaces of different surface finishes and glass probes of radius *R* = 17.97 mm with different surface roughnesses. Increasing the roughness of the glass probe from *ca.* 19 nm to 3.7 µm reduces the pull-off force against a smooth PDMS surface by *ca*. 67% from 27 to 9 mN. Against the fibrillar PDMS the situation is reversed and part of the adhesion from the ideal contact between smooth surfaces is restored in the contact with the rough glass probe. In the case of the smooth counterface, the introduction of fibres does not seem to benefit the contact.

Figure 6 indicates the effect surface texture had on the observed pull-off forces against both smooth and rough glass lenses. Using the smooth polymer slab, roughening the glass counterface reduced measured adhesion from about 27 mN with the smooth lens to values of *ca.* 9 mN, *i.e.*, a reduction of some 67%. The fibrillar PDMS surface had an even lower adhesive force measured against a smooth counterface at about 3 mN. On the other hand, adhesion between the rough glass surface and the fibrillar PDMS, at 17 mN, was very much more than with the smooth lens and nearly 63% of the value of adhesion between the two materials with smooth surfaces.

These results demonstrate that the interaction between adjacent contact spots that is responsible for the reduction in the overall adhesive force can be overcome, at least to some extent, if the softer surface exhibits an appropriate morphology to conform to the counterface topography. If individual adhered spots can be effectively isolated from their neighbors, for example by some form of compliant element, then the transfer of elastic energy from one region to another can be attenuated and adhesion maintained when the normal load is removed. When the fibrillar surface is loaded against a surface, smooth or rough, individual fibres, many of which are not perfectly normal to the surface, bend and buckle so acting as relatively weak, almost constant force, spring elements that store relatively little elastic energy. Consequently, when the surface is unloaded many of the small areas of intimate contact that have been formed, whether involving the tips or the flanks of the fibrils, remain intact and a level of macroscopic adhesion is maintained.

### Switchable Adhesion

**Figure 7:**
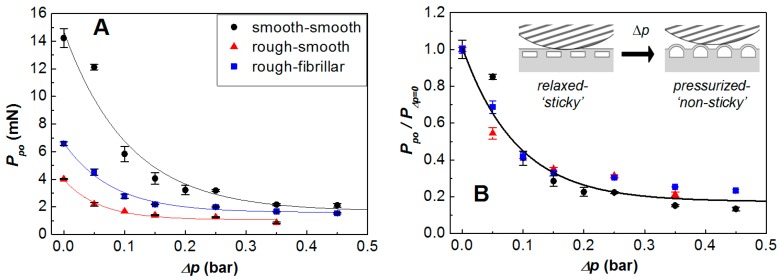
Changes in pull-off force with increased differential pressure in the subsurface channels. (**A**) Results from three different contact conjunctions: black circles correspond to the smooth glass probing lens against the specimen with smooth finish, red triangles to the sand blasted probe against the same smooth specimen and the blue squares to the roughened lens against a specimen with fibrillar texture, all specimens with same subsurface geometry; (**B**) Pull-off force normalized data by the maximum adhesion force, *i.e.*, at Δ*p* = 0, from each contact.

The presence of fibrils on the outer surface can be combined with sub-surface channels to retain some element of adhesive switchability by modifying the waviness (or long wavelength undulation) of the soft surface through pressurization of its subsurface structures in a similar manner as previously demonstrated for the contact between nominally smooth surfaces. This is illustrated by data shown in Figure 7A. Against both smooth and rough glass counterfaces the introduction of a positive differential pressure of the order of 0.2 bar in the subsurface features of a specimen (in this case channel widths of 300 µm and gaps between channels of 200 µm) significantly reduces the adhesion of the surface. Removal of the pressure restores adhesion to the previous level.

Data in Figure 7A are shown for experiments on specimens with each type of surface finish, smooth and fibrillar. The black circles show the results from a specimen with smooth surface measured against a smooth probe (*R_q_*~19 nm): these represent the control data set. The red triangles show the results from the same PDMS specimen with a smooth surface measured against the sand blasted probe (*R_q_*: 3.7 μm). The effect of the surface roughness is clearly observed in the reduction of about 70% of the adhesion of the surface in its relaxed state, *i.e.*, Δ*p* = 0, from ~14 to 4 mN. The introduction of the fibrillar texture on the polymer specimen surface led to enhanced adhesion of the specimen in its relaxed or “sticky” state against the rough probe with a restoration of about 50% of the pull-off force between smooth surfaces.

Figure 7B presents the data sets in Figure 7A normalized by the magnitude of adhesion from each surface in its relaxed state. A generalized reduction tendency of the adhesion force with increasing differential pressure can be described by the expression:

(3) $$P_{npo}=0.17+0.84\text{exp}\left(-∆p/0.09\right)$$

where *P*_npo_ is the normalized pull-off force and Δ*p* the differential pressure applied to the subsurface features. This expression is similar to that previously found for specimens of a range of subsurface geometries and smooth surface finish of $P_{npo}=0.032+0.985\text{exp}\left(-\Delta p/0.137\right)$ [1].

## 4. Conclusions

We show that adhesion between a smooth and rough hard surface and a softer counterface can be controlled by actively modifying the waviness of the softer surface using pneumatic activation of features just below the outer surface of the softer component. As with other examples of soft adhesion, any significant roughness on the higher modulus surface leads to a dramatic reduction in the magnitude of the pull-off force. It is possible to restore part of the adhesive effect by adding an appropriate fibrillar texture to the outer surface. When combined with active control of soft surface waviness, textured surfaces can achieve switchable adhesion with both smooth and non-smooth counterfaces. Conversely, stiffer surface features, for example conical pillars, can reduce adhesion. Examples of both effects can be found in nature.
